# SARS-CoV-2: Ultrastructural Characterization of Morphogenesis in an In Vitro System

**DOI:** 10.3390/v14020201

**Published:** 2022-01-20

**Authors:** Debora Ferreira Barreto-Vieira, Marcos Alexandre Nunes da Silva, Ana Luisa Teixeira de Almeida, Arthur da Costa Rasinhas, Maria Eduarda Monteiro, Milene Dias Miranda, Fernando Couto Motta, Marilda M. Siqueira, Wendell Girard-Dias, Bráulio Soares Archanjo, Patricia T. Bozza, Thiago Moreno L. Souza, Suelen Silva Gomes Dias, Vinicius Cardoso Soares, Ortrud Monika Barth

**Affiliations:** 1 Laboratório de Morfologia e Morfogênese Viral, Instituto Oswaldo Cruz, Fundação Oswaldo Cruz-Fiocruz, Rio de Janeiro 21040-900, RJ, Brazil; marquinhosans@gmail.com (M.A.N.d.S.); analuisaprovoc2012@gmail.com (A.L.T.d.A.); tukabr@gmail.com (A.d.C.R.); barth@ioc.fiocruz.br (O.M.B.); 2 Laboratório de Vírus Respiratórios e do Sarampo, Instituto Oswaldo Cruz, Fundação Oswaldo Cruz-Fiocruz, Rio de Janeiro 21040-900, RJ, Brazil; monteiro.meduarda@gmail.com (M.E.M.); milenediasmiranda@gmail.com (M.D.M.); fercoutomotta@gmail.com (F.C.M.); marildamts@gmail.com (M.M.S.); 3 Plataforma de Microscopia Eletrônica Rudolph Barth, Instituto Oswaldo Cruz, Fundação Oswaldo Cruz-Fiocruz, Rio de Janeiro 21040-900, RJ, Brazil; wendellbiom@gmail.com; 4 Núcleo de Laboratórios de Microscopia, Instituto Nacional de Metrologia, Qualidade e Tecnologia, Rio de Janeiro 25250-020, RJ, Brazil; braulioarchanjo@gmail.com; 5 Laboratório de Imunofarmacologia, Instituto Oswaldo Cruz, Fundação Oswaldo Cruz-Fiocruz, Rio de Janeiro 21040-900, RJ, Brazil; pbozza@gmail.com (P.T.B.); souzatml@gmail.com (T.M.L.S.); suelen.sgdias@gmail.com (S.S.G.D.); cardosodante42@gmail.com (V.C.S.); 6 Centro de Desenvolvimento Tecnológico em Saúde, National Institute for Science and Technology on Innovation on Diseases of Neglected Populations, Fundação Oswaldo Cruz-Fiocruz, Rio de Janeiro 21040-900, RJ, Brazil; 7 Programa de Imunologia e Inflamação, Universidade Federal do Rio de Janeiro (UFRJ), Rio de Janeiro 21941-901, RJ, Brazil

**Keywords:** SARS-CoV-2, Vero-E6 cells, ultrastructural studies, morphogenesis, transmission electron microscopy, scanning electron microscopy, 3D electron microscopy modeling

## Abstract

The pandemic caused by the severe acute respiratory syndrome coronavirus 2 (SARS-CoV-2) has impacted public health and the world economy and fueled a worldwide race to approve therapeutic and prophylactic agents, but so far there are no specific antiviral drugs. Understanding the biology of the virus is the first step in structuring strategies to combat it, and in this context several studies have been conducted with the aim of understanding the replication mechanism of SARS-CoV-2 in vitro systems. In this work, studies using transmission and scanning electron microscopy and 3D electron microscopy modeling were performed with the goal of characterizing the morphogenesis of SARS-CoV-2 in Vero-E6 cells. Several ultrastructural changes were observed—such as syncytia formation, cytoplasmic membrane projections, lipid droplets accumulation, proliferation of double-membrane vesicles derived from the rough endoplasmic reticulum, and alteration of mitochondria. The entry of the virus into cells occurred through endocytosis. Viral particles were observed attached to the cell membrane and in various cellular compartments, and extrusion of viral progeny took place by exocytosis. These findings allow us to infer that Vero-E6 cells are highly susceptible to SARS-CoV-2 infection as described in the literature and their replication cycle is similar to that described with SARS-CoV and MERS-CoV in vitro models.

## 1. Introduction

The severe acute respiratory syndrome coronavirus 2 (SARS-CoV-2), that is the causative agent of the coronavirus disease 2019 (COVID-19) pandemic [1], belongs to the *Coronaviridae* family (*Coronavirinae* subfamily) which includes four genera: *Alphacoronavirus, Betacoronavirus, Gammacoronavirus*, and *Deltacoronavirus*. While *Alphacoronavirus* and *Betacoronavirus* exclusively infect mammalian species, *Gammacoronavirus* and *Deltacoronavirus* have a wider host range that includes avian species [1,2]. The SARS-CoV-2 is grouped within the *Betacoronavirus* genus together with Middle East Respiratory Syndrome Coronavirus (MERS-CoV) and SARS-CoV, which are both also highly pathogenic, and HCoV-HKU1, HCoV-OC43, which causes seasonal and usually mild respiratory tract infections.

Coronaviruses are enveloped with positive-sense single-stranded RNA genome of 26–32 kb and have the largest genomes among RNA viruses. Phylogenetic analysis of SARS-CoV-2 revealed that is closely related (88–89% similarity) to SARS-like coronaviruses from bats—such as bat-SL-CoVZC45 (GenBank no. MG772933.1) and bat-SL-CoVZXC21 (GenBank no. MG772934.1)—and shares lower identity to SARS-CoV (~79% similarity) and MERS-CoV (~50% similarity) [3,4,5].

In vitro studies on the morphogenesis of coronaviruses demonstrated that the attached of these viruses to the host cell occurs from the interaction of the virus Spike protein (S protein) with the cellular ACE-2 (angiotensin converting enzyme 2) receptor [6,7,8]. Internalization of the particle occurs by endocytosis or through fusion between virus envelope and cellular membrane [9]. In SARS-CoV and MERS-CoV, the first moment of replication occurs on virus-induced double-membrane structures derived from the rough endoplasmic reticulum (RER), commonly referred to as ‘double-membrane vesicles’ (DMVs), located mostly in perinuclear areas [7,10,11]. These structures are also generated by other RNA viruses, such as Arteriviruses, Picornaviruses, and the Flaviviruses of genus *Hepacivirus* [12,13]. Viral RNA filaments inside DMVs were observed in studies in vitro with SARS-CoV-2, associating these compartments with viral replication [14].

Viruses with positive-sense single-stranded RNA genome replicate in host cell cytoplasm and induce intracellular membranous compartments harboring the sites of viral RNA synthesis. These replication factories are supposed to concentrate the components of the replicase and to shield replication intermediates from the host cell innate immune defense [15].

Given the fact that there is no therapeutic treatment for SARS-CoV-2 to date, studies in vitro are needed to understand the mechanism of infection and the virus–cell interaction, as well as to design effective strategies to inhibit virus morphogenesis. In this case, electron microscopy becomes an indispensable tool of analysis. The development of effective intervention strategies relies on the knowledge of molecular and cellular mechanisms of coronavirus infections, which highlights the significance of studying virus–host interactions at the molecular level to identify targets for antiviral intervention and to elucidate critical viral and host determinants that are decisive for the development of severe disease [16].

Vero cells, which are widely used in virus studies and in the production of human vaccines, have been shown to be a relevant model for studies of coronaviruses including SARS-CoV, SARS-CoV-2, and MERS-CoV [17,18,19,20,21,22,23].

In this work, we characterize the replicative cycle of SARS-CoV-2 from Vero-E6 lineage cells infected with a human clinical sample, using transmission electron microscopy and helium ion microscopy.

## 2. Materials and Methods

### 2.1. Virus Sample

A SARS-CoV-2, lineage B.1.1, was isolated from the nasopharyngeal swab from Brazilian patient by the National Influenza Centre (NIC) at Fiocruz, Rio de Janeiro. Total RNA was extracted from clinical sample using the QIAmp Viral RNA mini kit (Qiagen North Rhine-Westphalia, Hilden, Germany). Viral detection was done by real time reverse transcription polymerase chain reaction (RT-PCR) with TaqMan primers and probes (IDT) specific for the genes encoding the Envelope protein (E) and the viral RNA-dependent RNA Polymerase (RdRp), as described previously [2]. Reactions were performed with the Qiagen One Step RT-PCR kit (Qiagen, Germantown, MD, USA). Synthetic RNA sequences corresponding to E and RdRP targets [2] were used as positive controls. Putative coinfections in this sample were discarded by differential diagnosis analysis for other 17 respiratory viral pathogens and *mycoplasm pneumonieae*, as described previously [24]. Whole-genome sequences of isolate evaluated in this study are available in the Global initiative on sharing all influenza data (GISAID) under the accession numbers EPI_ISL_415105. For the experiments, the virus was amplified in Vero-E6 cells (African green monkey kidney). Virus titers were calculated by the tissue culture infectious dose at 50% (TCID_50_/mL) and the virus stocks kept in −80 °C freezer. All procedures were performed in a biosafety level 3 laboratory, according to WHO guidelines. The access to the genetic patrimony of the virus isolated is registered in Sistema Nacional de Gestão do Patrimônio Genético (SisGen ACCF49F). This research is approved by the Ethics Committee of Instituto Oswaldo Cruz (protocol number 2453470).

### 2.2. Cells and Virus Infection 

Prior to infection, Vero-E6 cultures were maintained in DMEM supplemented with 10% fetal bovine serum (FBS) and 100 U/mL of penicillin-streptomycin (1× Pen-Strep) and cultured at 37 °C and 5% CO_2_ [25]. All cell culture reagents were acquired from Gibco (Waltham, MA, USA). For infection, monolayers were washed twice with phosphate buffered saline (PBS), inoculated with multiplicity of infection of 0.01 diluted in non-supplemented DMEM and incubated for 1 h at 37 °C for virus adsorption. After adsorption time, the inoculum was removed and cells were kept at 37 °C in DMEM supplemented with 2% FBS and 1× PenStrep. Non-infected control cultures (mock) were prepared using pure non-supplemented DMEM as infected cells. Monolayers were inspected daily under light microscope for observation of cytopathic effect (CPE), until 72 h post infection (pi). All procedures were performed in a biosafety level 3 laboratory, according to WHO guidelines.

### 2.3. Transmission Electron Microscopy (TEM) and Helium Ion Microscopy (HIM)

For transmission electron microscopy analysis, the infected and non-infected control (mock) monolayers of cells were tripsinized at 48 and 72 h pi. Cell suspensions were fixated in 2.5% glutaraldehyde in sodium cacodylate buffer (0.2 M, pH 7.2), post-fixated in 1% buffered osmium tetroxide, dehydrated in acetone, embedded in epoxy resin, and polymerized at 60 °C over the course of three days [26,27]. Ultrathin sections (50–70 nm) were obtained from the resin blocks. The sections were picked up using copper grids (300 mesh and no coating) and observed using Cs Corrected FEI Titan 80–300 (ThermoFisher Scientific, Waltham, MA, USA) and Hitachi HT 7800 (Hitachi, Tokyo, Japan) transmission electron microscopes. In the FEI Titan images were collected in scanning transmission electron microscopy (STEM) mode using a high-angle annular-dark field detector (HAADF) working at 80 kV high tension, 19 mrad convergence angle and probe size around 0.1 nm, 100 pA beam current, 2 up to 8 μs dwell time, and 2048 × 2048 pixels per image. For analysis in helium ion microscopy, infected and non-infected control (mock) monolayers were grown on sterile glass coverslips and fixed at 48 and 72 h pi, in 2.5% glutaraldehyde in sodium cacodylate buffer (0.2 M, pH 7.2), dehydrated in ethanol and submitted to critical-point-drying. The cells were analyzed in an Orion NanoFab Helium Ion microscope (Zeiss, Baden-Württemberg, Oberkochen, Germany) equipped with a flood gun and an Everhart–Thornley secondary electrons detector, flood gun is used to allow analyzing the cells without any conductive coating. Images were collected at 30 kV high tension, around 0.8 pA beam current, 32 up to 256-line average, 1 µs dwell time, 100 µs flood gun time, and 2048 × 2048 pixels per image.

### 2.4. Focused Ion Beam Scanning Electron Microscopy (FIB-SEM) and 3D Modeling

FIB-SEM image acquisition was carried out with a Zeiss Auriga crossbeam electron microscope (Zeiss, Baden-Württemberg, Oberkochen, Germany). The epoxide-embedded sample (block) of Vero-E6 cells 48h pi with SARS-CoV-2 prepared for TEM as described above was mounted on a support stub, coated with a 10 nm layer of gold and transferred to the microscope chamber. The sample was tilted 54° towards the ion column in order for the block surface to be oriented perpendicularly to the Ga+ ion beam. The area of interest was exposed by FIB milling of a U-shaped trench. Milling conditions were 30 kV acceleration voltage and a beam current of 16 nA (for coarse milling) and 2 nA for polishing. FIB slicing was performed at 20 nm thickness with an ion beam current of 1 nA. Imaging of the block surface was carried out at 1.8 kV using a backscattered electron detector and 5.4 nm pixel size.

The image series were aligned and the structures of interest were manually segmented for 3D models production using the open source software IMOD [28].

### 2.5. Measurement of Virus Particle Size

The images were collected in a Zeiss Orion Nanofab helium ion microscope. The particle size statistical analysis was performed using ImageJ software. Each virus particle was selected manually and deleted from the images, after thresholding images they were converted into binary and then only virus particles were selected and counted using the command particle analysis from the menu analysis. About 600 virus particles were analyzed and the average size and standard deviation were extracted.

### 2.6. Immunofluorescence Staining

The cells were stained as previously described [29,30]. In short, Vero-E6 cells were seeded in coverslips and after 48 and 72 h of infection, were fixated using 3.7% formaldehyde. Cells were rinsed three times with PBS containing 0.1 mM CaCl_2_ and 1 mM MgCl_2_ (PBS/CM) and then permeabilized with 0.1% Triton X-100 plus 0.2% BSA in PBS/CM for 10 min (PBS/CM/TBSA). Cells were stained with rabbit polyclonal antibody anti-spike antibody (#56578—NOVUSBIO) at 1:250 dilution for overnight, followed by a rabbit anti-IgG Dylight 550 at 1:1000 dilution for 1 h. The coverslips were mounted in slides using an antifade mounting medium (VECTASHIELD^®^, Burlingame, CA, USA). Nuclear recognition was based on DAPI staining (1 μg/mL) for 5 min. Fluorescence was analyzed by fluorescence microscopy with an 100× objective lens (Olympus, Tokyo, Japan).

## 3. Results

Analysis of Vero-E6 cells infected with SARS-CoV-2 under inverted light microscopy demonstrated a CPE, that was mostly evident from 48 h pi. The CPE appeared as rounding and detaching of cells and formation of syncytia (data not shown).

### 3.1. Ultrastructural Cellular Change Caused by SARS-CoV-2 Infection

**Figure 1:**
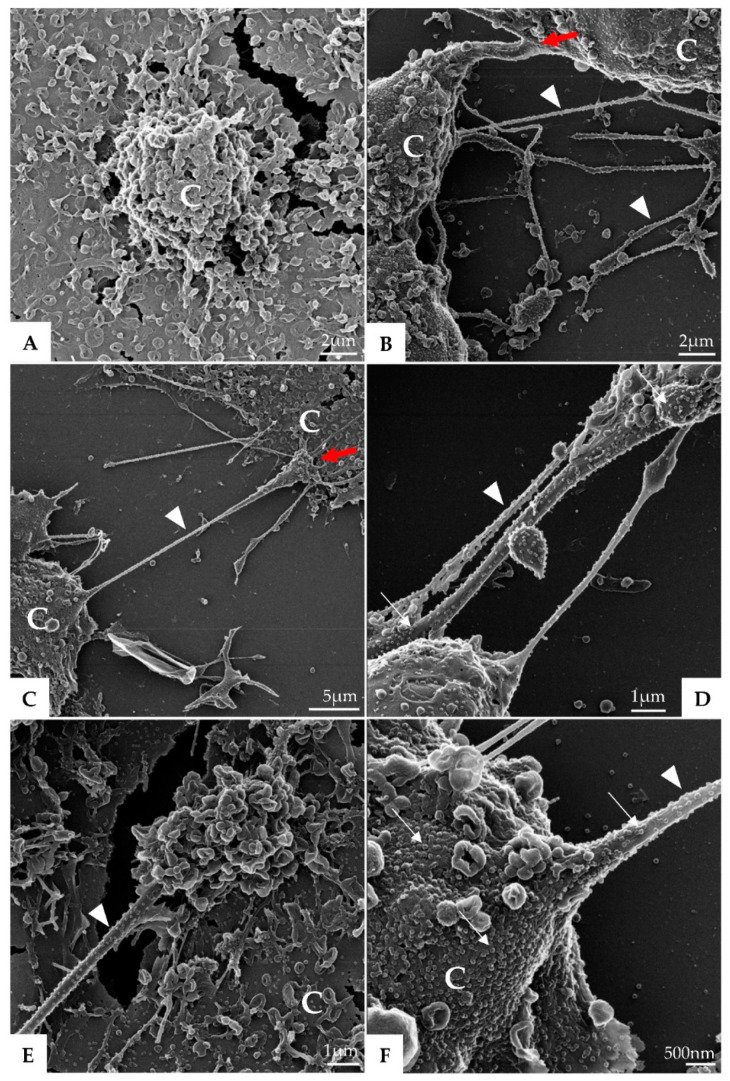
Vero-E6 cells 48 h post-infection with SARS-CoV-2 (HIM images). (**A**) Uninfected Vero-E6 cell at 48 h of cultivation (cell control). (**B**–**F**) Infected cells presenting filopodia (arrowhead); connection (red arrow) between adjacent cells mediated by filopodia was observed. Virus particles (thick arrow) attached to cell filopodia and with cell membrane were observed. Cell (C).

**Figure 2:**
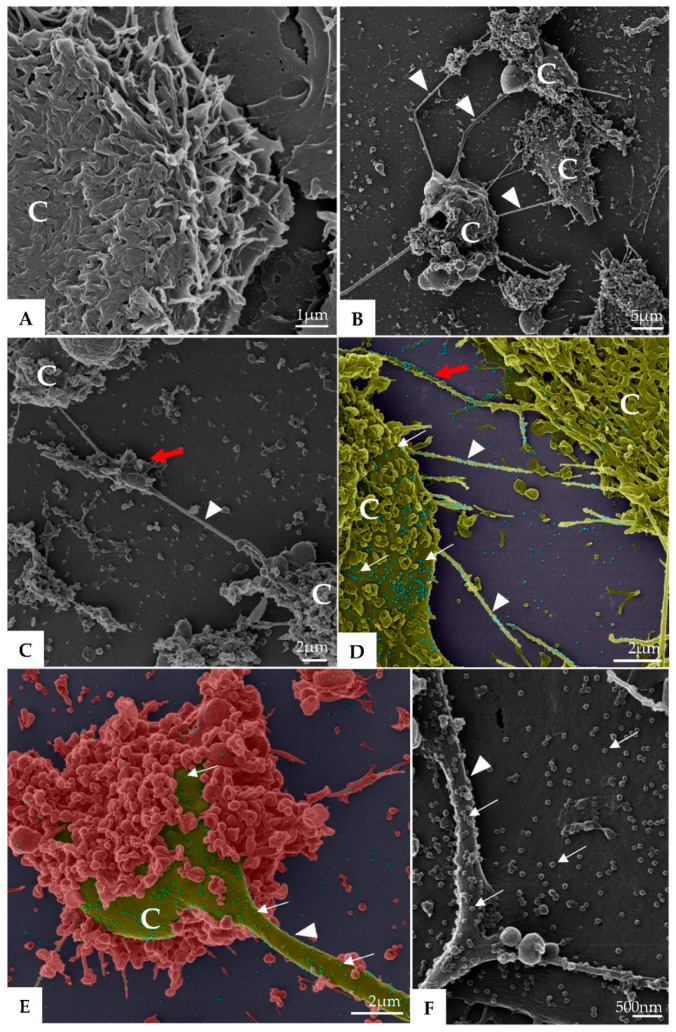
Vero-E6 cells 72 h post-infection with SARS-CoV-2 (HIM images). (**A**) Uninfected Vero-E6 cell at 72 h of cultivation (cell control). (**B**–**E**) Infected Vero-E6 monolayer, connection (red arrow) between cells mediated by filopodia was observed (**B**–**D**). Virus particles (thick arrow, blue structures) was detected attached to cell filopodia (green, image (**D**)) and with cell membrane (green or red, image (**D**–**F**). Cell (C), filopodia (arrowhead). The images (**D**,**E**) were colored in Adobe photoshop.

**Figure 3:**
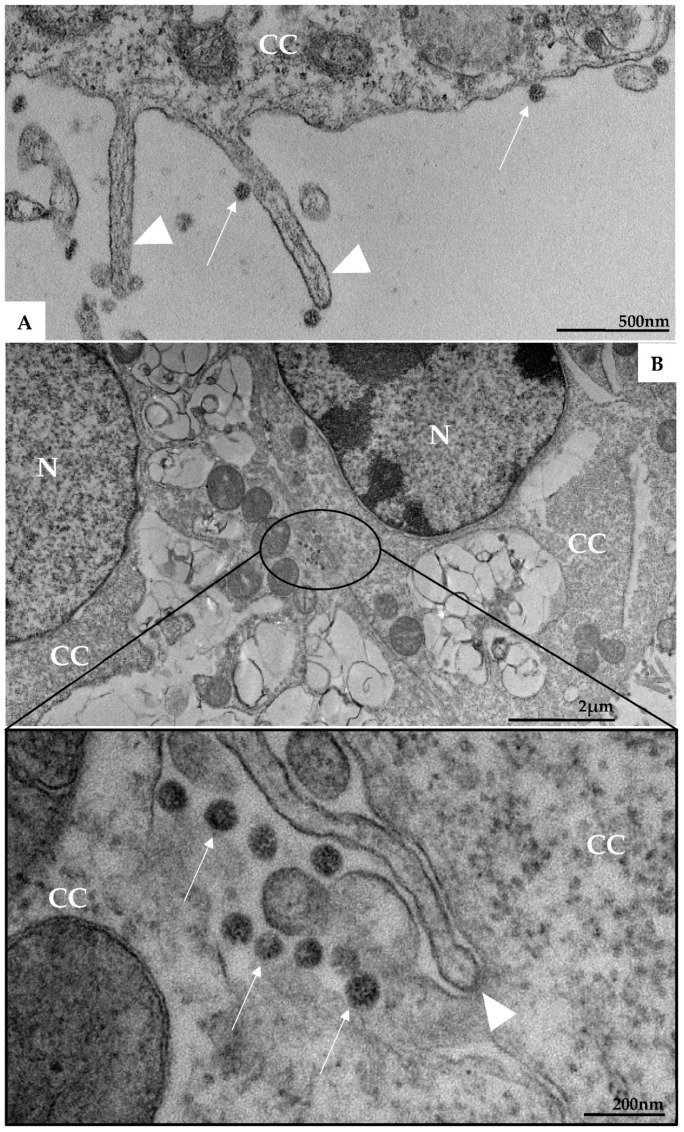
Microvilli in Vero-E6 cells 48 h post-infection with SARS-CoV-2 (TEM images). Microvilli (arrowhead) presenting adsorbed SARS-CoV-2 particles (thick arrow) (**A**). Interaction between adjacent cells mediated by Microvilli (**B**). Microvilli (arrowhead), cell cytoplasm (CC), nucleus (N).

**Figure 4:**
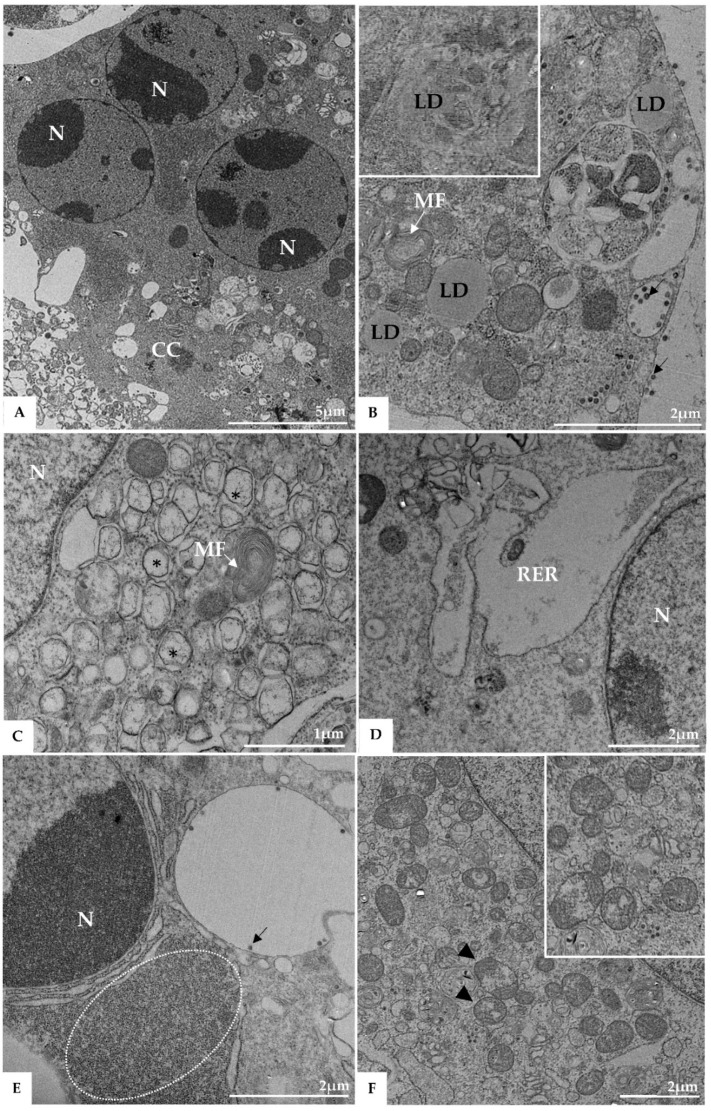
Syncytia formation (**A**), proliferation of lipid droplets (LD) (**B**), and double membrane vesicle (*) (**C**), myelin figures (MF) (**B**,**C**), thickening of the rough endoplasmic reticulum (RER) (**D**), electron-dense ribosomes (marked area) (**E**), and degeneration of mitochondria (arrowhead) (**F**) in Vero-E6 cells 48 h post-infection with SARS-CoV-2 (TEM images). Virus particles (arrow), cell cytoplasm (CC), nucleus (N).

Morphological analysis of Vero-E6 cells at 48 and 72 h pi by using of MET and HIM showed changes associated to SARS-CoV-2 infection, including: (I) Cell activation—Cell activation was evidenced by a marked plasmatic membrane projection (filopodia and microvilli) (Figure 1B–F and Figure 2B–F), connection between adjacent cells mediated by these filopodia (Figure 1B–C, Figure 2B–D, and Figure 3B), viral particles associated with filopodia and microvilli (Figure 1B–F, Figure 2B–F, and Figure 3A) and to the cell membrane (Figure 1B–F and Figure 2B–F). Filopodia, as well as any changes in cell topography, were not observed in non-infected Vero-E6 cells at 48 (Figure 1A) or 72 h (Figure 2A) of cultivation. (II) Syncytia—Syncytial formation (multinucleated cells resulting from cell fusion) in Vero-E6 cells monolayer infected with SARS-CoV-2 were commonly observed as shown in the Figure 4A. (III) Lipid droplets (LD) accumulation (Figure 4B). (IV) Proliferation of double membrane vesicles (DMV). These vesicles were observed throughout the cytosol (Figure 4C). (V) Numerous myelin figures (Figure 4B,C). (VI) Thickening of the RER (Figure 4D), increased number of more electron-dense ribosomes (Figure 4E), and mitochondrial swelling and vacuolation (Figure 4F) were also observed.

### 3.2. SARS-CoV-2 Particles Diameter and Morphology

**Figure 5:**
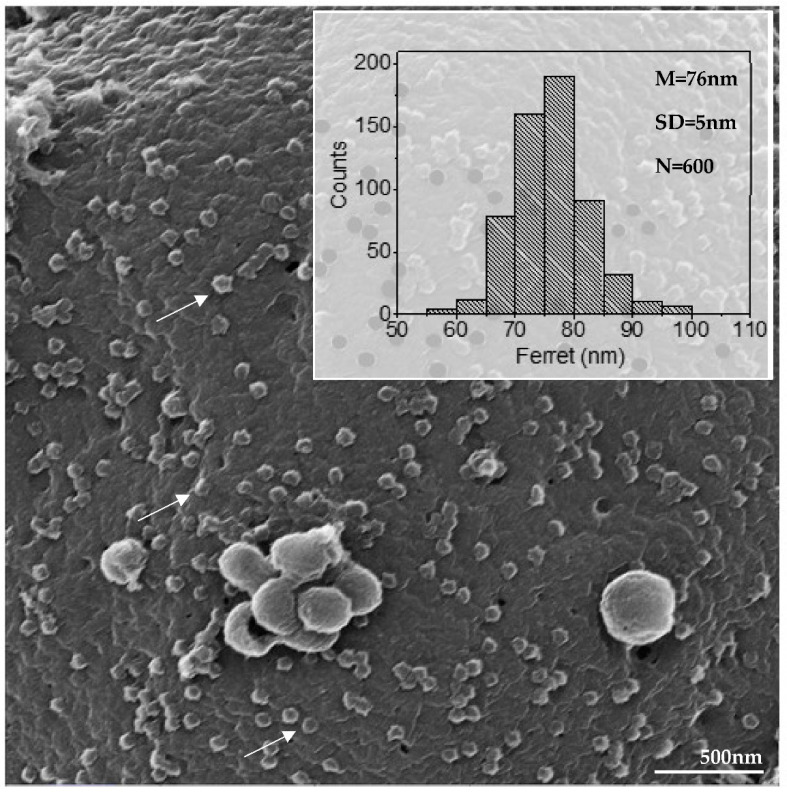
Diameter of SARS-CoV-2 particles attached in cell membrane (arrow) (HIM image). The average diameter of the SARS-CoV-2 particles was around 76 nanometers. Mean (M), standard deviation (SD), number of measured particles (N), virus particles (arrow).

**Figure 6:**
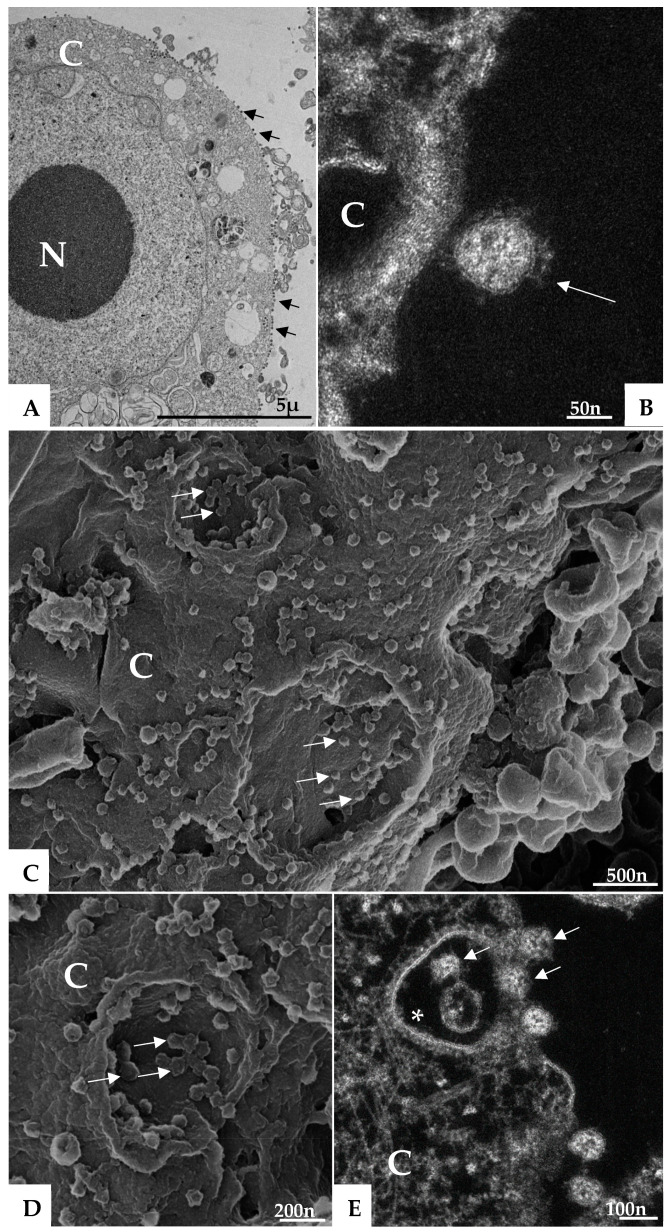
Attachment and endocytosis of SARS-CoV-2 particles (TEM (**A**), HAADF-STEM (**B**,**E**) and HIM (**C**,**D**) images). SARS-CoV-2 particles (arrows) attached to the cell membrane (**A**–**D**) and being internalized by endocytosis (arrow) (**E**). Endocytic vesicles coated in clathrin (*) (**E**). Cell cytoplasm (CC), cell membrane (CM), nucleus (N).

Morphometric analysis by helium ion microscopy of SARS-CoV-2 particles attached on the Vero-E6 cell membrane showed that their mean diameter was 76 nanometers (Figure 5). MET showed particles with a spherical morphology exhibiting spikes on their envelopes (Figure 6B).

### 3.3. Morphogenesis of SARS-CoV-2 in Vero-E6 Cell

**Figure 7:**
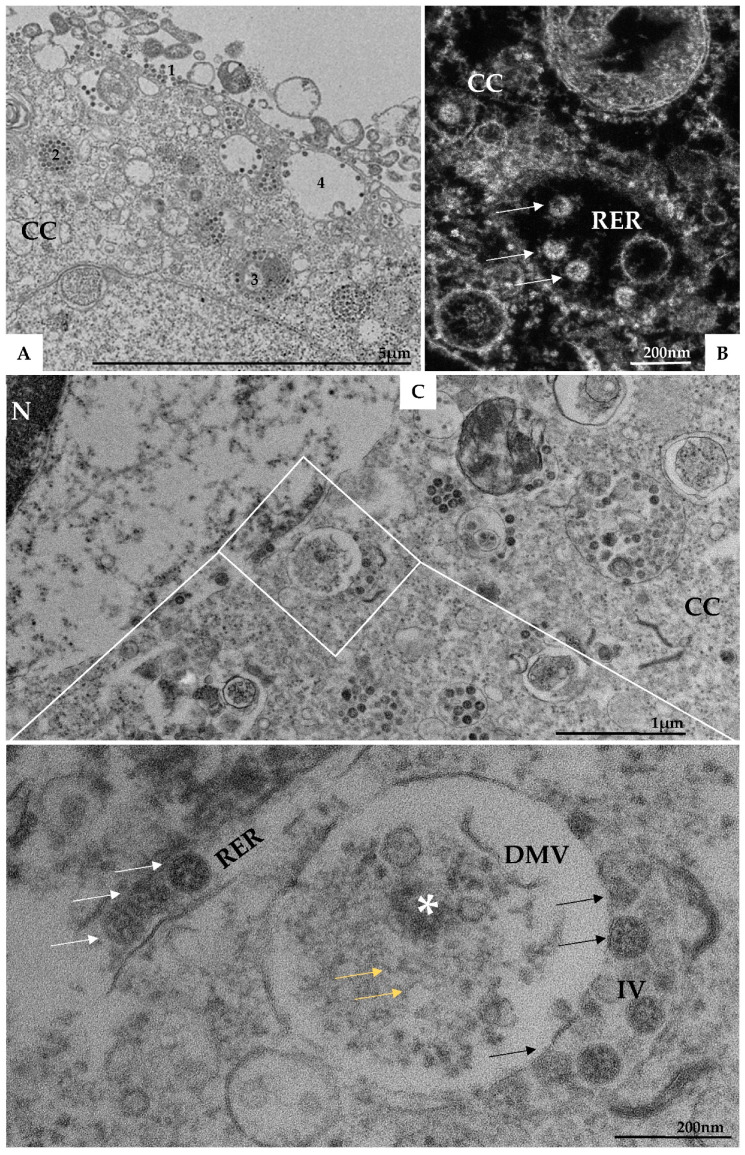
SARS-CoV-2 particles in lumen of several cellular compartments. (**A**) SARS-CoV-2 particles attached to the cell membrane (1), on endosomes (2), on the rough endoplasmic reticulum (3 and image (**B**) (arrow)), and being exocytosed via vesicles (4). (**C**) Presence of double membrane vesicle (DMV) containing in their lumen electron-dense material (*) and RNA-like filaments (yellow arrow). Budding of viral particles (black arrow) from these vesicles to into intermediate vesicles (IV) was observed. Virions were observed in rough endoplasmic reticulum cisterns (white arrow). Cell cytoplasm (CC), nucleus (N), and rough endoplasmic reticulum cisterns (RER). Transmission electron microscopy.

**Figure 8:**
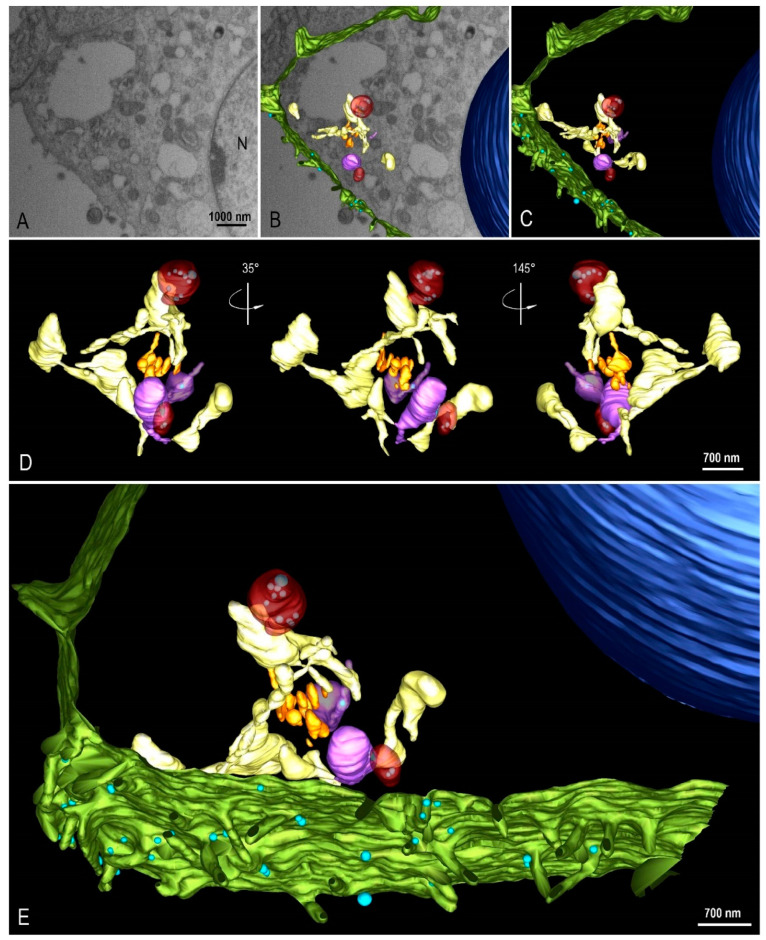
3D modeling of Vero-E6 48 h post-infection with SARS-CoV-2. (**A**) FIB-SEM image of the block surface showing an infected cell. (**B**,**C**) Combination of a block surface image and the 3D model of structures of interest. (**D**) Different angle views of a region of interest showing the organization and interaction between different cellular compartments. (**E**) 3D model showing a global view of the model. Green (cell membrane), light blue (virus particle), dark blue (nucleus), yellow (rough endoplasmic reticulum), orange (Golgi complex), red (double membrane vesicle), purple (intermediate vesicle). See video in Supplementary Material (Video S1).

(I) Virus particle entry—Many virus particles attached to the cell surface (adsorption) (Figure 6A–E) was observed. The entry of the SARS-CoV-2 virus particles into the cells was most commonly realized by clathrin coated endocytic vesicles (Figure 6E), although the fusion of the virus envelope with the cell membrane has been observed also (data not shown).

(II) Assembly of viral particles—Virus particles have been observed inside several cytoplasmic compartments including endosomes, RER, DMVs, intermediate vesicles (IV), and exosomes (Figure 7A,B). FIB-SEM images and three-dimensional (3D) modeling of the Vero-E6 cell, 48h pi, showed the interaction between different cellular compartments (Figure 8A–E see also Video S1).

**Figure 9:**
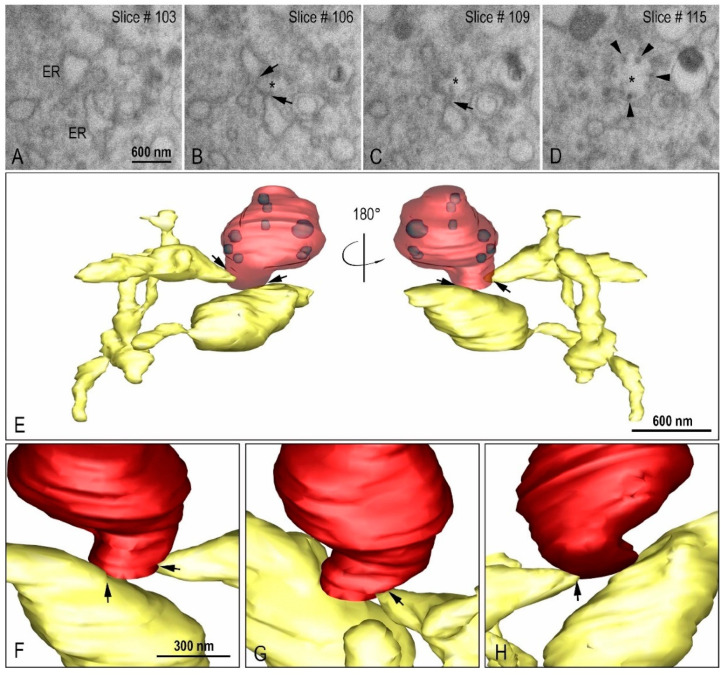
Interaction between the rough endoplasmic reticulum (ER) and double membrane vesicle (DMV). (**A**–**D**) FIB-SEM image sequence showing the ER and its connection (arrows) to a DMV (asterisk). Some virus particles can be seen inside the DMV (arrow heads). (**E**–**H**) 3D model of the DMV (red), virus particles (purple) inside the DMV and the ER (yellow). The regions of connection of the ER to the DMV are indicated with arrows. Interconnections within the ER network can be observed.

**Figure 10:**
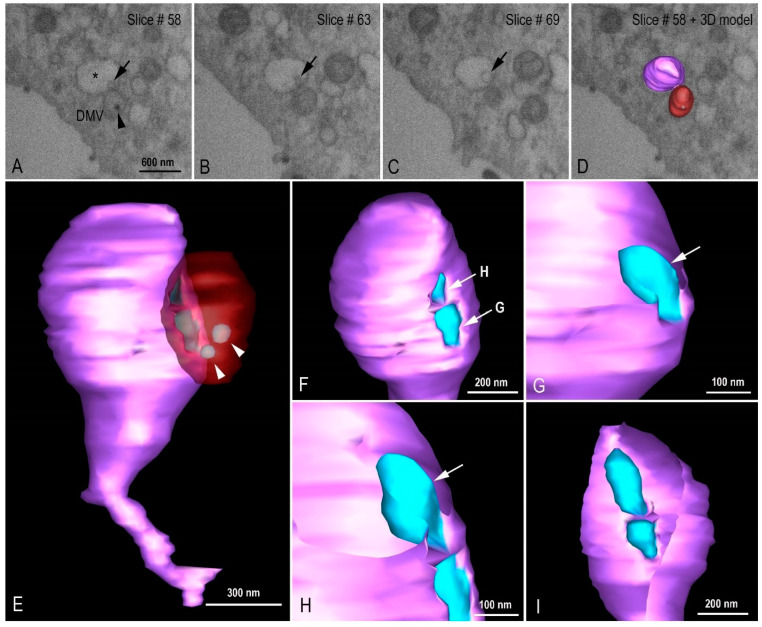
Virion budding events at intermediate vesicle (IV). (**A**–**D**) FIB-SEM image sequence exhibiting different virus budding stages (arrows) at IV (asterisk) and the proximity with a double membrane vesicle (DMV). Virus particles can be observed inside the DMV (arrow head). (**E**–**I**) 3D model showing the DMV (red with transparence), virus particles (light blue, arrow heads), and the IV (purple). (**F**) Extra vesicular view of two virus particle budding regions. The arrows indicate the cutting plane showed in G and H. (**G**) Initial stage of virus particle budding. (**H**) Final stage of virus particle budding. (**I**) Intra vesicular view of the virus budding. See video in Supplementary Material (Video S2).

Using transmission electron microscopy (Figure 7C) and three-dimensional reconstruction (Figure 9A–E) the DMVs were observed located close to the RER, suggesting that DMVs result from remodeling of the RER membranes. Inside the DMVs, accumulation of electron-dense material, RNA-like filaments were observed (Figure 7C). IVs associated with these DMVs were observed as well as the presence of virus particles budding from the DMVs membranes into IVs lumen, suggesting that part of the assembly of viral particles can occur in this location (Figure 7C). The budding of the viral particles from the DMV into IV was also demonstrated in three-dimension reconstruction (Figure 10A–I, see also Video S2).

**Figure 11:**
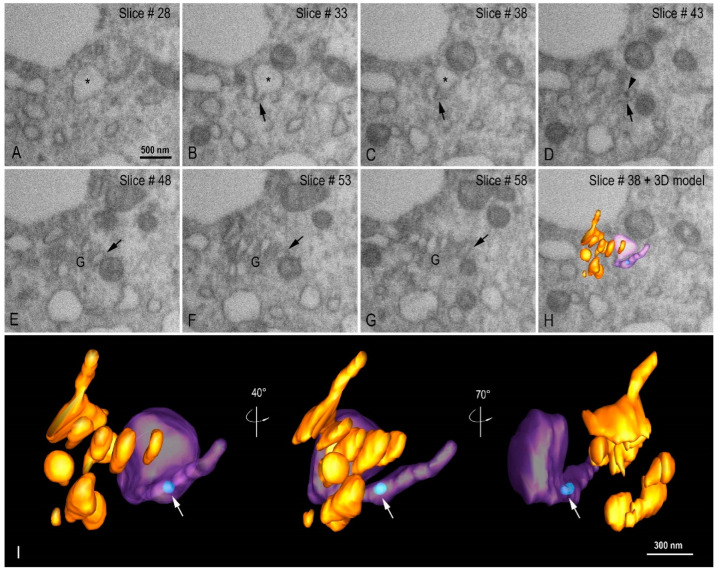
Organization of the Golgi complex (G) and intermediate vesicle (IV). (**A**–**H**) FIB-SEM image sequence showing an IV (asterisk) with a tubular region (arrow) in close proximity with to the Golgi complex. A virus particle can be seen inside the tubular region of the IV (arrow head). (**I**) 3D model of the G (orange), IV (purple in transparence), and a virus particle (light blue, arrow) at different angle views. See video in Supplementary Material (Video S3).

Golgi complex was observed by FIB-SEM image sequence and 3D modeling (Figure 11A–I, see also Video S3) close to the IVs containing virus particles, a fact that allows us to infer a possible relationship between both.

**Figure 12:**
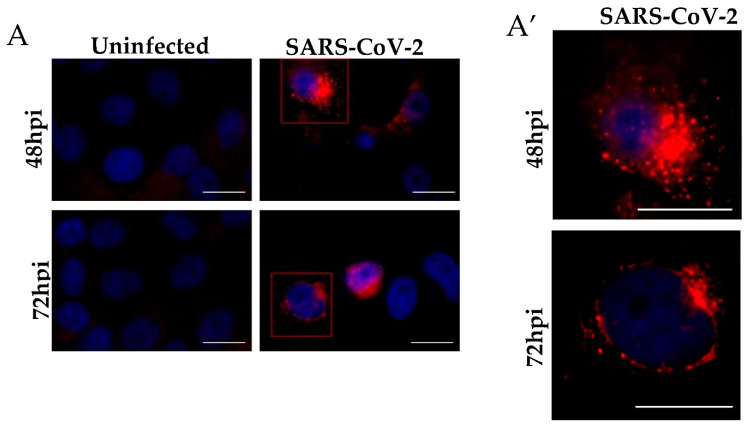
Immunofluorescence analyses of Vero-E6 cells after SARS-CoV-2 infection for 48 and 72 h. (**A**) Uninfected cells with 48 and 72 h of cultivation. (**A′**) Representative zoom images. The virus was detected by the presence of the spike protein (red) performed by immunofluorescence, and cell nuclei stained with DAPI (blue). Hours post-infection (h pi). Scale bar 20 μm.

**Figure 13:**
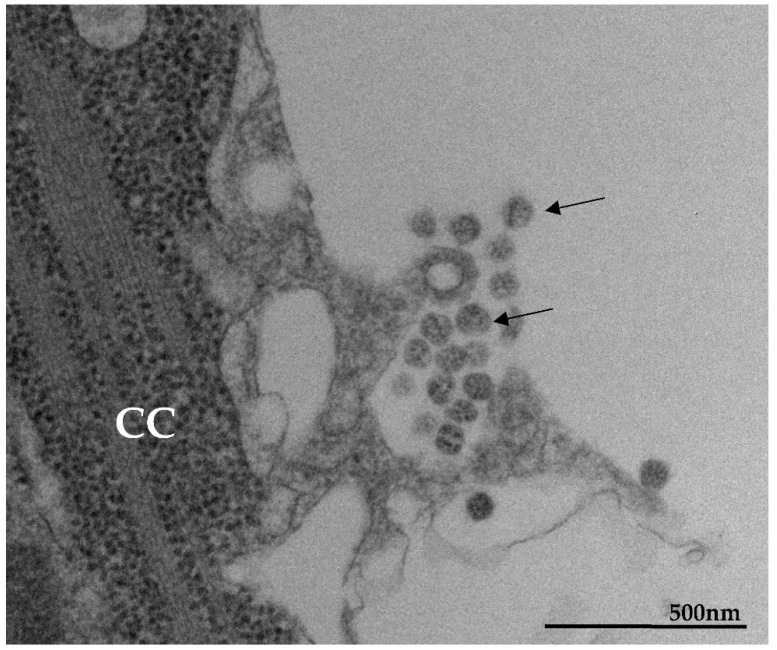
Extrusion of SARS-CoV-2 particles (MET). The particles released by exocytosis. Cell cytoplasm (CC), virus particles (arrow).

Immunolabeling tests using the immunofluorescence technique of Vero-E6 cells demonstrated the detection of SARS-CoV-2 Spike protein diffused throughout the cytosol both at 48 (Figure 12B,F) and 72 h pi (Figure 12D,H), suggesting viral replication and spreading infection. Detection of Spike protein of SARS-CoV-2 were not observed in the uninfected cells with 48 (Figure 12A,E) and 72 (Figure 12C,G) h of cultivation.

(III) Extrusion of virus particles. Virions were detected in multiple vesicles at the cell periphery and the release of the particles occurred by exocytosis (Figure 13). Fusion of the exocytic vesicle membrane with the cell cytoplasmic membrane was observed.

## 4. Discussion

TEM, STEM, HIM, and 3D modeling were used in the present study aiming to characterize the main virus-induced cellular changes as well as the replicative cycle of SARS-CoV-2 in Vero-E6 cells infected with a Brazilian clinical sample.

We observed topographic changes in Vero-E6 cells at 48 and 72 h pi by SARS-CoV-2 evidenced by a marked cellular activation by the presence of numerous filopodia with associated viral particles. Connection between adjacent cells mediated by these filopodia was also observed. We believe that the role of filopodia in this in vitro model is associated with infection maximization. In vitro studies with Vero cells with SARS-CoV and SARS-CoV-2 corroborate our findings [23,31,32]. Enveloped viruses can spread by two different routes: the cell-free aqueous environment or by cell-to-cell contact [33,34,35]. Cell-to-cell contact spreading has unique advantages. The transfer speed is greater because the replication cycle of release, transmission, and entry can proceed in a faster manner. Another advantage of cell-to-cell contact is immune evasion because the limited exposure in extracellular space avoids interaction with neutralizing antibodies. Lastly, by exploiting cell-to-cell communication, the physical and immunological barriers can be overcome to spread the infection [34,35]. Viruses can hijack the filopodia system for their own use during the life cycle under the following strategies: viral surfing (the binding of virus to filopodia induced a rapid lateral movement toward the cell body), filopodial retraction (the filopodia on virally infected cells pull the virus inward), and filopodial bridges to assist virus transport between the cells [35,36].

Syncytial formation in Vero-E6 cells monolayer 48 and 72 h pi with SARS-CoV-2 were commonly observed in our analysis. In ultrastructural studies of Vero cells infected with SARS-CoV multinucleated syncytial cells were occasionally seen [37]. Studies with patients with COVID-19 showed lungs contain infected pneumocytes with abnormal morphology and frequent multinucleation [38]. The authors infer that generation of these syncytia results from activation of the SARS-CoV-2 spike protein at the cell plasma membrane level.

Our analysis demonstrated accumulation of LDs in infected cells, which is corroborated by a previous study conducted by our group [29]. In the above-mentioned study, in vitro SARS-CoV-2 infection were seen to modulate pathways of lipid synthesis and uptake as monitored by testing for CD36, SREBP-1, PPARγ, and DGAT-1 expression in monocytes and triggered LD formation in different human cell lines. Ultrastructural analysis by transmission electron microscopy of Vero-E6 cells infected with SARS-CoV-2 showed viral particles colocalizing with LDs, suggesting that LDs might serve as an assembly platform. Indeed, inhibition of LD formation in Vero-E6 lead to decreased SARS-CoV-2 replication [29]. Our data are also corroborated with the findings of Nardacci et al. (2021) that demonstrated that SARS-CoV-2 infection induce the accumulation of LDs, both in cultured cells and in type II pneumocytes of lung from infected patients. In addition, LDs were often observed in close contact with swollen mitochondria and may participate in mechanisms of cytopathogenesis [39].

SARS-CoV-2 particles attached to the cell surfaces with an average diameter of 76 nanometers and with spherical morphology exhibiting spikes on their envelope, which is characteristic of viruses belonging to the *Coronaviridae* family. This data is very consistent with previous in vitro studies of SARS-CoV [9] and SARS-CoV-2 in which the virion diameter ranged from 60 to 140 nm [14,38,40,41,42].

We observed that the entry of the SARS-CoV-2 into the host cell occurs most commonly by endocytosis, although the fusion of the virus envelope with the cell membrane has also been observed. This is consistent with our previous ultrastructural studies with SARS-CoV-2 [23] and others from literature with SARS-CoV and MERS-CoV which support both mechanisms [9,43,44].

In the present study, DMVs located close to the RER were observed, suggesting that these vesicles result from remodeling of the RER membranes. Presence of RNA-like filaments and electron-dense material inside the lumen of DMVs was observed. DMVs are induced during the replication of a variety of RNA viruses [45,46,47,48] and were identified as the sole compartment where viral RNA transcription occurs for coronaviruses [49]. Morphological analysis of different cell lines infected with SARS-CoV, MERS-CoV, and SARS-CoV-2—as well as in our studies—has demonstrated that the DMVs are derived from the RER. In the studies concerning SARS-CoV and MERS-CoV, the nonstructural proteins 3 and 4 were predominantly responsible for orchestrating this process [7,50,51,52]. In some of these models, the RER lumen constitutes the space between the DMV’s inner and outer membrane, while the enclosed space is of cytoplasmic origin and enriched in double-stranded RNA (dsRNA) [14].

Our data corroborates the findings from the SARS-CoV and MERS-CoV studies, as well as the analysis with Vero-E6 and human pulmonary cell lines (Calu3) infected with SARS-CoV-2, in which DMVs containing viral RNA inside were found [14,23,32]. Wolff et al. [47], using cellular cryoelectron microscopy, identified a molecular pore complex spanning both membranes of the DMVs, which interconnects the interior of the vesicles with the cytoplasm and would allow the release of newly synthesized RNA from the DMV interior into the cytoplasm.

We observed IVs associated with DMVs, as well as the presence of virus particles budding from the DMVs membranes into IVs lumen, suggesting that part of the assembly of particles can occur in this localization. Close proximity of IVs with the Golgi complex was observed, a fact that allows us to infer a possible relationship between IVs and the Golgi complex. Knoops et al. [7] and Siu et al. [53] observed large assemblies of convoluted membranes, often in close proximity to DMV clusters in Vero-E6 cells infected with SARS-CoV. The authors suggest that these structures are probably identical to the “reticular inclusions” that were firstly observed in cells infected with mouse hepatitis coronavirus [54] and were later referred to as ‘clusters of tubular cisternal elements’, which may have a connection to the endoplasmic reticulum–Golgi intermediate compartment (ERGIC) [55]. The structural proteins membrane (M), nucleocapsid (N), envelope (E), and S—together with the genomic RNA—drive the assembly of new virus particles, which in the case of other coronaviruses bud into ERGIC [56].

We observed virions in multiple smooth vesicles at the cell periphery, close to the cell membrane. Fusion of membrane of the smooth vesicles with the cell membrane resulting in the release of the particles was also observed, too. This mechanism of viral progeny release was detected in our previous studies with SARS-CoV-2 [24], and corroborates data presented by Qian et al. [57] and Qinfen et al. [9] from in vitro studies with alveolar type II cells and Vero-E6 cells infected with SARS-CoV.

In our immunolabeling tests by the immunofluorescence technique, the robust presence of Spike protein was observed in all Vero-E6 cell cytoplasms at both 48 and 72 h pi, proving the high permissiveness of this cell line to SARS-CoV-2 infection, as demonstrated in our ultrastructural studies.

Our analyses demonstrated how essential electron microscopy is when it comes to the analysis of a causative agent of an unknown disease, a situation already experienced in 2003 with SARS-CoV [58], Ebola [59,60,61], Hendra [62], Nipah [63], and monkeypox [64].

This study showed that the Vero-E6 cell is a good in vitro model for studies of SARS-CoV-2 biogenesis and that it can be used for pre-clinical studies.

## Data Availability

Not applicable.

## Supplementary Materials

The following supporting information can be downloaded at: https://www.mdpi.com/article/10.3390/v14020201/s1, Video S1. Three-dimensional reconstruction of Vero-E6 48 h post-infection with SARS-CoV-2. Combination of a FIB-SEM image sequence and the 3D model presenting different cellular compartments engaged in SARS-CoV-2 replication. Green (cell membrane), light blue (virus particle), dark blue (nucleus), yellow (rough endoplasmic reticulum), orange (Golgi complex), red (double membrane vesicle), purple (intermediate vesicle). Video S2. Three-dimensional reconstruction of SARS-CoV-2 particles budding events at intermediate vesicle. 3D model showing different virus budding stages and the proximity with a double membrane vesicle. Red with transparence (double membrane vesicle), light blue (virus particles), purple (intermediate vesicle). Video S3. Three-dimensional reconstruction of organization of the Golgi complex and intermediate vesicle. Combination FIB-SEM image sequence and the 3D model showing an intermediate vesicle with a tubular region in close proximity with the Golgi complex. A virus particle can be seen inside the tubular region of the intermediate vesicle. Orange (Golgi complex), purple in transparence (intermediate vesicle), light blue (virus particle).
